# Alterations in Adenylate Nucleotide Metabolism and Associated Lipid Peroxidation and Protein Oxidative Damage in Rat Kidneys Under Combined Acetaminophen Toxicity and Protein Deficiency

**DOI:** 10.3390/antiox15010105

**Published:** 2026-01-13

**Authors:** Oksana M. Voloshchuk, Halyna P. Kopylchuk, Maria S. Ursatyy, Karolina A. Kovalchuk, Oleksii Skorokhod

**Affiliations:** 1 Department of Biochemistry and Biotechnology, Yuriy Fedkovych Chernivtsi National University, 2 Kotsiubynskoho St., 58012 Chernivtsi, Ukraine; g.kopilchuk@chnu.edu.ua (H.P.K.); ursatyi.mariia@chnu.edu.ua (M.S.U.); kovalchuk.karolina.a@chnu.edu.ua (K.A.K.); 2 Department of Life Sciences and Systems Biology, University of Turin, via Accademia Albertina 13, 10123 Turin, Italy

**Keywords:** acetaminophen APAP, nutritional protein deficiency, metabolic stress, kidneys, adenylate nucleotides, AMP deaminase, cytosolic 5′-nucleotidase, lipid peroxidation, reduced protein SH-groups, protein carbonyls

## Abstract

Acetaminophen (APAP) overdose is a major cause of acute liver failure and can be fatal, often without early symptoms. Protein deficiency, arising from illness or inadequate diet, impairs growth, immunity, and tissue repair. Both conditions can harm the kidneys, yet the impact of energy imbalance on renal physiology remains unclear. In this study, APAP toxicity and a low-protein diet induced behavioral suppression and tissue damage, as evidenced by reduced whole-body, liver, and kidney weights in rats. In kidney mitochondria of rats exposed to only toxic APAP doses, ATP levels declined sharply while ADP and AMP increased. AMP deaminase and ATPases’ activities rose about twofold and 1.5-fold, respectively, whereas cytosolic 5′-nucleotidase activity fell nearly threefold, suggesting compensatory responses to disrupted energy balance. The strongest reductions in ATP and the greatest increases in AMP and ATPase activity occurred in APAP-intoxicated rats fed a low-protein diet. This combination also intensified lipid peroxidation and oxidative protein damage, evidenced by elevated TBARS, reduced protein SH-groups, and increased protein carbonyls. Overall, APAP intoxication with protein deficiency disrupts renal energy metabolism, leading to mitochondrial dysfunction and structural kidney injury. Nutritional status therefore critically influences drug-induced nephrotoxicity, and antioxidant strategies may help prevent damage under metabolic stress.

## 1. Introduction

Acetaminophen (N-acetyl-para-aminophenol, APAP) overdose is a leading cause of acute liver failure. Without prompt treatment, it can result in severe hepatotoxicity or death even though early symptoms may be mild or absent. On the other hand, the human diet plays a crucial role in maintaining quality of life, especially in regions affected by geopolitical instability or natural events that lead to food scarcity. Protein deficiency, whether stemming from illness or poor dietary intake, can significantly impair growth, immune defense, and tissue regeneration, posing a serious health challenge. Both APAP overdose and protein deficiency are known contributors to kidney dysfunction. However, the specific mechanisms by which energy imbalance affects key renal physiological processes under conditions of APAP-induced toxicity and protein deficiency remain not fully understood.

The functional activity of healthy kidneys largely depends on the energy supply to their cells. The production of sufficient ATP is crucial for maintaining cellular anabolism and physiological processes such as reabsorption and the regulation of water-salt and acid-base balance [1]. Adequate energy supply is essential for kidney cell recovery after injury, and cellular energy levels are considered a marker of cell viability [2]. Renal tubular epithelial cells rank among the most energy-demanding cell types in the body, and reductions in ATP levels contribute to increased apoptosis and dedifferentiation [3]. In recent years, particular attention has been paid to energy metabolism disorders in the pathophysiology of kidney diseases [4]. Kidney damage is accompanied by a rapid decline in energy production, depletion of energy resources, and epithelial cell death [5]. Mitochondrial dysfunction in the kidneys, especially disruptions in oxidative phosphorylation, leads to impaired reabsorption and filtration capacity, which underlies the pathogenesis of various nephropathies [6]. Renal tubular cells expend the majority of their ATP on active reabsorption processes, highlighting the pivotal role of mitochondria in sustaining normal kidney function [7].

The primary pathway for ATP synthesis in proximal tubules is oxidative phosphorylation. Although glucose-derived acetyl-CoA primarily contributes to the Krebs cycle, other substrates such as fatty acids, amino acids, and lactate also support mitochondrial energy production. Under normal conditions, the kidneys have a limited ability to metabolize glucose for energy, a limitation that becomes more pronounced during disease states [8]. Consequently, alternative energy sources, including those derived from proteins, may play a vital role in sustaining renal cellular function.

Therapeutic targets have increasingly focused on the disrupted energy metabolism and mitochondrial dysfunction that characterize acute kidney injury. Nevertheless, despite the potential for therapies aimed at restoring metabolic balance and mitigating tissue damage, the mechanisms underlying the energy dysregulation that alters key renal physiological processes during APAP overdose and dietary protein deficiency still require extensive investigation. It is known that acute APAP overdose can cause potentially fatal acute kidney injury, but the exact mechanism of APAP-induced kidney damage is still not fully clarified. Metabolites of APAP, particularly N-acetyl-p-benzoquinone imine (NAPQI) or p-aminophenol, are known to have a high affinity for binding to kidney cell proteins and depleting glutathione reserves, thereby enhancing nephrotoxicity [9]. It has also been shown that reduced dietary protein intake decreases kidney mass and impairs glomerular filtration, leading to damage and inflammation of the renal tubules [10]. Therefore, studying the relationship between dietary protein availability in animals with APAP intoxication and the energy status of kidney cells is important for understanding the genesis and mechanisms of progression of such disorders. It may also be useful for exploring prevention or treatment strategies that reduce damage or promote kidney function recovery [11].

Adenine nucleotide concentrations, adenosine 5′ triphosphate, diphosphate, and monophosphate (ATP, ADP, and AMP), may serve as key indicators of a cell’s energy status. Evolutionarily, they play a central role in cellular metabolism by linking energy-producing pathways with energy-consuming processes, thereby maintaining metabolic balance and supporting vital cellular functions. The ratio and content of ATP, ADP, and AMP in cells depend on energy demand and expenditure, with AMP and ADP acting as positive effectors that stimulate catabolic processes. It is known that the mechanism of maintaining the adenylate pool in most tissues is closely linked to AMP metabolism [12], making changes in AMP levels a particularly informative marker of cellular energy-metabolic status.

Two cytosolic enzymes are particularly important for maintaining adenine nucleotide balance: 5′-nucleotidase (Enzyme Commission nomenclature EC 3.1.3.5) and AMP deaminase (AMPD, EC 3.5.4.6). These enzymes mediate the metabolic conversion of purine nucleotides and regulate intracellular levels of key modulators such as AMP, adenosine, and inosine [13,14,15]. AMPD catalyzes the hydrolytic deamination of AMP to inosine monophosphate (IMP), removing the amino group from the C6 position of the adenine ring. This reaction is essential for adenine nucleotide catabolism and helps regulate AMP levels during high-energy demand or metabolic stress. By lowering AMP concentration, AMPD contributes to maintaining the adenylate energy charge (AEC) and cellular energy homeostasis [14]. In this context, the ATP:AMP ratio is important because it is a very sensitive indicator of the cell’s energy status [15]. In the kidney, AMPD initiates the purine nucleotide cycle, which prevents complete purine degradation and generates fumarate and ammonia, supporting nitrogen balance and metabolic flexibility. Additionally, AMPD activity influences adenosine synthesis by modulating AMP availability [16]. 5′-Nucleotidase (5′-NT) catalyzes the hydrolysis of the 5′-phosphoester bond of ribonucleoside 5′-monophosphates, producing the corresponding ribonucleoside and inorganic phosphate. Its primary physiological function is the conversion of adenosine monophosphate (AMP) to adenosine, a key step in purine catabolism and in the generation of adenosine as an extracellular signaling molecule [17]. Adenosine acts as a critical endogenous regulator in the renal system, influencing key physiological processes such as renin release, glomerular filtration rate, and renal vascular tone. It is also a fundamental mediator of the tubuloglomerular feedback mechanism. Adenosine levels rise significantly during negative energy balance, when ATP hydrolysis exceeds ATP synthesis [18]. Such changes can contribute to nephropathology [19], as observed in this study’s model of toxicity-induced kidney damage.

Direct enzyme damage caused by oxidative stress and lipid peroxidation products, whose increased formation has been reported in kidneys under acetaminophen intoxication [20,21], may underlie the observed here enzymatic activity imbalance. This imbalance appears to occur in parallel with organelle membrane injury, particularly within mitochondria. Various enzyme dysregulations induced by oxidation- and lipid-peroxidation-derived products have been demonstrated in numerous conditions [22,23,24,25], but more extensive research is needed to clarify these mechanisms in renal pathology and toxicity-induced kidney injury.

Because oxidative stress and lipid peroxidation drive both enzyme dysfunction and mitochondrial membrane damage, antioxidants may help interrupt this cascade. By reducing ROS and limiting the formation of peroxidation products such as 4-hydroxynonenal (4-HNE), they can preserve adenylate balance and maintain mitochondrial energy metabolism. This targeted protection suggests that antioxidants could meaningfully ameliorate the metabolic disturbances observed under intoxication-induced renal injury.

The aim of this study is to comprehensively evaluate the physiological responses, adenylate nucleotide levels (ATP, ADP, AMP), activities of key energy-metabolism enzymes, and the extent of lipid peroxidation and oxidative protein damage in the renal tissue of rats subjected to APAP-induced intoxication. These assessments were conducted under varying dietary protein regimens to explore the potential modulatory effects of nutritional status on purine metabolism and mitochondrial function during toxic injury.

## 2. Materials and Methods

Unless otherwise specified, all reagents were of molecular biology grade and purchased from Merck Sigma-Aldrich (St. Louis, MO, USA).

### 2.1. Animals and Experimental Protocols

All animal procedures were approved by the Institutional Ethical Comity of Yuriy Fedkovych Chernivtsi National University, Ukraine (Protocol No. 3, 1 October 2024) and conducted in accordance with national and international guidelines for the care and use of laboratory animals: European Convention for the Protection of Vertebrate Animals Used for Experimental and Other Scientific Purposes (Strasbourg, 1986), as well as the recommendations of the Bioethical Review of Preclinical and Other Scientific Research Conducted on Animals (Kyiv, Ukraine, 2006).

Experiments were conducted on male white outbred rats weighing 130–140 g and aged 99–100 days. Animals were housed in groups of three per cage with ad libitum access to water. Housing conditions were maintained at a controlled temperature of 20 ± 1 °C, with filtered air, 60–70% relative humidity, and a 12 h light/dark cycle [26]. Prior to the start of the study, rats were acclimated to the laboratory environment for 7 days, during which they had free access to a standard diet and water under the same light/dark cycle. At the start of the experiment, 36 animals were allocated into four groups (9 animals per group, which is the standard number used in our animal studies). The control group consisted of intact rats maintained for four weeks on a complete semi-synthetic diet containing 14% protein (casein), 10% fat, 10% sucrose, and a mineral-vitamin mix according to the American Institute of Nutrition recommendations [27]. The second group (LPD) received an isoenergetic low-protein diet (4.7% protein) for 29 days. The third group (APAP) was maintained on the complete diet and subjected to acute acetaminophen-induced toxicity for two days (see details below). The fourth group (LPD/APAP) was fed the low-protein diet as the LPD group for 29 days and exposed to APAP toxicity during the last two days [28]. Feed intake was monitored daily throughout the study. The pre-weighed food was provided each morning, and the remaining amount was weighed after 24 h to calculate the average daily consumption per animal. No significant differences in total food intake were observed between the control and experimental groups, ensuring that the results were due to the dietary composition rather than caloric restriction. At day 129 of life, at the conclusion of the experiment, all animals were humanely euthanized for tissue collection by decapitation under light ether anesthesia, in accordance with the European Convention for the Protection of Vertebrate Animals (1986) and European Union Council Directive 2010/63/EU. This method was selected to allow rapid tissue collection and to minimize potential interference of anesthetic agents with mitochondrial respiratory chain enzymes. Immediately after confirmation of death, kidneys were rapidly excised and processed at ice-cold temperature.

### 2.2. Acetaminophen Toxicity Induction

Acute toxic injury in experimental animals was induced by oral administration of acetaminophen (APAP) at a dose of 1250 mg/kg body weight. The drug was delivered by intragastric gavage as a suspension in a 2% starch gel solution at a fixed volume of 1 mL per rat. The APAP–starch gel mixture was thoroughly homogenized to obtain a uniform suspension and administered using a stainless steel gavage needle, ensuring complete delivery of the calculated dose to each animal. Treatment was administered once daily to 127-day-old rats during the final two days prior to sample collection. This dosage and timeframe have been previously described as sufficient to induce APAP toxicity in experimental models [29,30].

### 2.3. Animal Welfare Monitoring and Clinical Scoring

In this study, animal health and behavior were monitored daily using a structured clinical scoring system based on established animal welfare guidelines. Clinical assessments were conducted each day between 9:00 and 11:00 a.m. Each rat was evaluated for key parameters, including spontaneous activity (apathy), fur condition, and motor function (body posture and coordination). Scores were recorded in a dedicated laboratory clinical log. To ensure objectivity, observations were performed independently by two researchers who were not involved in dietary preparation or APAP administration. Cages were labeled with neutral numerical codes, and observers were blinded to experimental group assignments. Qualitative observations were evaluated using a semi-quantitative ordinal scale (0–3), with 0 indicating normal physiological status and 3 indicating severe impairment.

### 2.4. Histopathological Examination of Renal Tissues

Harvested kidneys were rinsed twice with cold phosphate-buffered saline (PBS) and then fixed in 10% formaldehyde for histopathological examination. Paraffin blocks were prepared, and 5 µm thick sections were obtained and stained with hematoxylin and eosin (H&E) using standard procedures. All H&E-stained kidney sections from the different experimental groups were evaluated histologically in a blinded manner using a Leica DM IRB microscope equipped with a DFC420C camera and DFC software (version 3.3.1; Leica Microsystems, Wetzlar, Germany). Histological alterations, including edema, inflammatory cell infiltration, and tissue damage, were assessed using a semi-quantitative scoring system.

### 2.5. Isolation of Mitochondria

Intact mitochondria were isolated from subcellular kidney fractions using a conventional method, as previously described [26,31]. Briefly, the mitochondrial fraction was obtained by differential centrifugation at 4 °C. The homogenization medium consisted of 10 mmol/L Tris(hydroxymethyl)aminomethane (Tris), 1 mmol/L EDTA, 0.5 mmol/L DTT and 250 mmol/L sucrose, adjusted to pH 7.4. Tissue homogenates were first spun at 600× *g* for 10 min at 4 °C to sediment nuclei, unbroken cells, and large membrane fragments. The resulting supernatant was then subjected to a spin at 10,000× *g* for 10 min, yielding the crude mitochondrial pellet. The pellet was washed in homogenization medium by resuspension and a second centrifugation at 10,000× *g* for 10 min at 4 °C. The final mitochondrial pellet was re-suspended in 1 mL of homogenization medium. To ensure reproducibility of the extraction and enable valid comparisons between conditions, mitochondrial protein concentration was quantified by measuring protein content with the Bradford assay after lysing a small aliquot in Laemmli buffer. Experiments with mitochondrial ATPases were performed immediately after the isolation of intact mitochondria.

### 2.6. Isolation of Cytosolic Fraction

The cytosolic fraction was isolated using a standard subcellular fractionation method [32]. All procedures were conducted at 0–4 °C to preserve sample integrity. Sodium orthovanadate (0.5 mM) was added only to samples in which adenine nucleotides were quantified, in order to inhibit P-type ATPases and preserve the true in vivo ATP concentration. Kidney homogenates were subjected to differential centrifugation to separate subcellular components. Sequential centrifugation steps were performed at 1000× *g*, 2000× *g*, 5000× *g*, and 10,000× *g* for 10 min each. The resulting supernatant was then centrifuged at 21,000× *g* for 60 min to pellet the microsomal fraction. The final supernatant was collected as the cytosolic fraction and used for analyses.

### 2.7. Quantification of Adenine Nucleotides (AMP, ADP, ATP)

The content of adenine nucleotides (AMP, ADP, ATP) was studied in cytosolic fraction using thin-layer chromatography on Silufol UV-254 plates [2]. Free nucleotides were extracted from the mitochondrial fraction using 0.8 M HClO_4_ for 30 min at 0–4 °C. Protein-free perchlorate extracts were obtained by centrifugation for 15 min at 1500 g. Supernatants were neutralized with K_2_CO_3_ to pH 7.0 and centrifuged again under the same conditions. Aliquots of the supernatant were applied to chromatographic plates. After separation of adenine nucleotides in a mobile phase consisting of dioxane, isopropanol, water, and ammonia (4:2:4:1), they were quantified by direct spectrometry. Nucleotide spots were visualized under UV light and eluted with 0.1 M HCl for 20 min [2]. Absorbance of the eluate was measured at 260 nm by spectrophotometer Agilent 8453E (Agilent Technologies, Santa Clara, CA, USA). Data are presented as AMP, ADP, and ATP concentrations, as well as ATP/ADP and AMP/ATP ratios. The adenylate energy charge (AEC) was calculated using the formula: AEC = ([ATP] + 0.5 × [ADP])/([ATP] + [ADP] + [AMP]).

### 2.8. Determination of AMP Deaminase Activity

AMP deaminase (AMPD; EC 3.5.4.6) activity in the cytosolic fraction was determined by a spectrophotometric method based on measuring the ammonia generated during AMP deamination. The assay, including controls, was performed under four conditions: Reagent blank, Standard, Control, and AMPD test. The control and reaction mixtures were prepared as follows: (i) Reagent blank: 420 μL of phosphate buffer (50 mM NaH_2_PO_4_ and Na_2_HPO_4_·12H_2_O, pH 6.5); (ii) Standard: 400 μL of 75 μM ammonium sulfate standard solution and 20 μL of distilled water; (iii) Control: 380 μL of buffered AMP solution (21 mM AMP in 50 mM phosphate buffer, pH 6.5); (iv) AMPD test: 380 μL of buffered AMP solution and 40 μL of the cytosolic fraction from experimental sample. All tubes were thoroughly mixed and incubated at 37 °C for 60 min to allow the enzymatic reaction to proceed. After incubation, 1200 μL of phenol-nitroprusside solution (106 mM phenol, 0.17 mM sodium nitroprusside) and 1200 μL of alkaline hypochlorite solution (11 mM NaOCl, 125 mM NaOH) were added to all tubes. At this point, 40 μL of the cytosolic fraction was also added to the control tube to account for background absorbance in a condition without reaction time. All tubes were then thoroughly mixed and incubated again at 37 °C for 30 min to allow color development. Absorbance was measured at 630 nm against distilled water by spectrophotometer Agilent 8453E (Agilent Technologies, Santa Clara, CA, USA). AMPD activity was expressed as micromoles of NH_3_ released per mg of protein per minute.

### 2.9. Determination of 5′-Nucleotidase Activity

The activity of 5′-nucleotidase was determined using a previously described method based on the quantitative measurement of inorganic phosphate (Pi) released during the enzymatic hydrolysis of AMP [13]. The assay was performed under two conditions: (i) Control condition, designed to measure non-specific alkaline phosphatase activity, included 0.2 mL of the cytosolic fraction, 0.1 mL of 0.02 M manganous sulfate (MnSO_4_), 1.3 mL of 40 mM barbitone buffer (pH 7.5), and 0.2 mL of 0.1 M nickel chloride (NiCl_2_), which acts as a selective inhibitor of 5′-nucleotidase; (ii) Test condition, designed to measure total enzyme activity, included 0.2 mL of the cytosolic fraction, 0.1 mL of 0.02 M MnSO_4_, and 1.5 mL of 40 mM barbitone buffer (pH 7.5). Both tubes were pre-incubated at 37 °C, and the reaction was initiated by adding 0.2 mL of 10 mM AMP. The mixtures were incubated at 37 °C for 30 min. The reaction was terminated by adding 2 mL of 10% trichloroacetic acid (TCA), followed by mixing and brief standing before centrifugation. For colorimetric determination of Pi, 2 mL of the supernatant from each tube was collected and additional controls were performed: for blank and phosphate standard preparations, 1 mL of distilled water and 1 mL of phosphate standard solution, respectively, were mixed with 1 mL of 10% TCA. To each of the four tubes (control condition, test condition, phosphate standard and blank), 3 mL of 2 M acetate buffer (pH 4.0), 0.5 mL of 5% ammonium molybdate, and 0.5 mL of metol reagent (prepared by dissolving 2 g metol (p-methylaminophenol sulfate) and 10 g sodium sulfite in water to a final volume of 100 mL) were added and mixed. The chromogenic reaction was allowed to develop for 10 min, and absorbance was measured spectrophotometrically at 680 nm. Specific 5′-nucleotidase activity was calculated by subtracting the non-specific alkaline phosphatase activity from the total activity. Enzymatic activity was expressed as nanomoles of Pi released per milligram of protein per minute.

### 2.10. Determination of Mitochondrial ATPases’ Activity

The activity of mitochondrial ATPases was determined by measuring the accumulation of Pi following ATP hydrolysis by isolated mitochondria. Mitochondria were assumed to be intact in control samples and damaged/leaky under experimental conditions. The incubation mixture contained 400 µmol Tris-HCl buffer (pH 7.4), 5 µmol disodium ATP, 7.5 µmol MgSO_4_, 0.01 µmol 2,4-dinitrophenol, 7.5 µmol CaCl_2_, 120 µmol NaCl, and 20 µmol KCl. The reaction was initiated by adding 50 µL of mitochondrial suspension containing 1 mg of total protein and incubated at 37 °C for 15 min. Pi content was measured colorimetrically [33,34]. Oligomycin was used at 1 µM to specifically inhibit F_o_F_1_-ATPsyntase. ATPase activity was expressed as nanomoles of Pi released per milligram of protein per minute.

### 2.11. Detection of Lipid Peroxidation and Protein Oxidative Damage

Lipid peroxidation in mitochondria was measured using the Thiobarbituric Acid Reactive Substances (TBARS) assay, which primarily detects malondialdehyde (MDA), along with other aldehydic byproducts of lipid peroxidation such as acrolein and 4- HNE [35,36]. TBARS concentration was determined by its reaction with 2-thiobarbituric acid (TBA) at 95 °C in an acidic solution for 20 min, forming a pink chromogen. The absorbance of this complex was measured spectrophotometrically at 532 nm (ε = 1.56 × 10^5^ M^−1^·cm^−1^) [36]. Results were expressed as nanomoles of TBARS per milligram of protein.

Protein carbonylation was assessed by measuring dinitrophenylhydrazone derivatives formed through the reaction of oxidized amino acid residues with 2,4-dinitrophenylhydrazine (DNPH) [37,38,39]. After derivatization, proteins were precipitated using trichloroacetic acid (TCA) and washed with ethanol to remove excess DNPH. The resulting protein pellet was resuspended in 6 M guanidine hydrochloride to solubilize the DNP-derivatized proteins. The absorbance of the solubilized complex was measured spectrophotometrically at 375 nm. Results were expressed as nanomoles of carbonyl protein derivatives per milligram of protein.

The content of protein sulfhydryl (–SH) groups was determined using Ellman’s reagent (5,5′-dithiobis-(2-nitrobenzoic acid), DTNB), which reacts with free thiol groups to form a mixed disulfide and 2-nitro-5-thiobenzoate (TNB), a yellow-colored anion. The amount of TNB produced, measured spectrophotometrically at 412 nm, is directly proportional to the concentration of free thiol groups in the sample [40]. The concentration of free –SH groups was calculated using a molar extinction coefficient of 11.4 × 10^3^ M^−1^·cm^−1^ and expressed as nanomoles per milligram of protein [40,41].

### 2.12. Statistical Analysis

All data are presented as median and interquartile range or as the mean of nine independent measurements ± standard error of the mean (SEM). Overall differences among groups were assessed using a Kruskal–Wallis test: H parameter and significance p are presented. Pairwise post hoc comparisons were performed using two-sided Mann–Whitney U tests with Holm correction for multiple testing by IBM SPSS Statistics for Windows (version 29.0; IBM Corp., Chicago, IL, USA). Significance of differences is indicated for all presented data and is considered significant at *p* ≤ 0.05.

## 3. Results

### 3.1. The Effect of Toxic Doses of APAP and a Low-Protein Diet on Rat Behavior

This study began with observations of the behavior of rats subjected to toxic doses of APAP and a low-protein diet. Excluding the healthy control group, all experimental groups exhibited a general suppression of behavior, characterized by reduced spontaneous activity (apathy), impaired motor function (abnormal body posture and coordination), and a dull or poor fur appearance. These observations were subsequently quantified using a semi-quantitative scoring system. The results are summarized in Table 1 as medians with interquartile ranges. Overall differences among groups were assessed using the Kruskal–Wallis test, with corresponding H statistics and *p* values reported. Significant differences were observed among groups (*p* < 0.01). Complete statistical parameters are provided in the Supplemental Materials.

Signs of apathy, impaired fur condition, and altered motor coordination were observed in the LPD, APAP, and LPD/APAP groups compared with the Control group (Table 1). Notably, these disturbances were more pronounced in the APAP group than in the LPD-only group. A synergistic effect of the low-protein diet and APAP exposure was evident in the LPD/APAP group, in which all assessed parameters were strongly increased compared with the APAP group. For example, comparing the mean values, the fur condition worsening score increased by 2.2 ± 0.5-fold, and the General Clinical Score increased by 1.6 ± 0.4-fold. Although these behavioral changes are nonspecific, they were significant (see Table 1 for *p* values) and reflect systemic toxic damage, with the most pronounced effects observed in the LPD/APAP group.

### 3.2. Liver and Kidney Weight Alterations

As the next step, we assessed whether morphological parameters, specifically liver and kidney weights, were affected by toxic doses of acetaminophen in the APAP group, by diet in the LPD group, and by the combination of both factors in the LPD/APAP group (Table 2). Overall differences among groups were assessed using the Kruskal–Wallis test, with corresponding H statistics and *p* values reported. Complete statistical parameters, including the significance of differences between all pairs of groups, are provided in the Supplemental Materials.

We observed that animals maintained on a low-protein diet (groups LPD and LPD/APAP) exhibited a growth delay phenotype, as evidenced by significantly lower body weights. At the time of measurement, the body weights of animals in the respective groups were reduced by 19.5 ± 6.6% and 20.0 ± 6.5% compared to the control group, as shown in Table 2. The overall body weight of the APAP group did not show significant changes, indicating that APAP toxicity alone did not affect general growth under standard dietary conditions.

**Figure 1:**
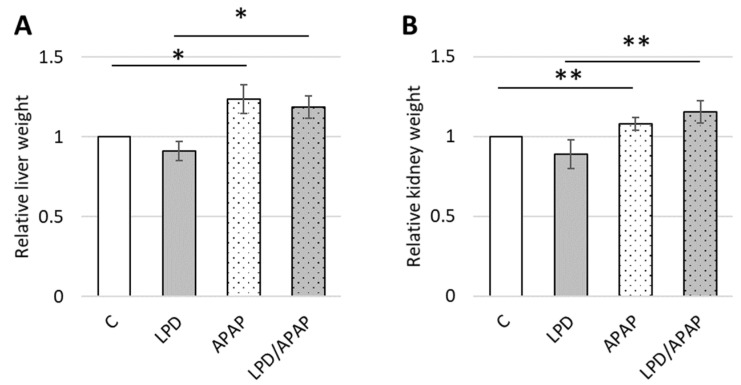
Organ weight alterations in low-protein diet and APAP toxicity. Relative liver weight (**A**) and relative kidney weight (**B**), normalized to the corresponding values in control animals, are shown for rats from four experimental groups: control animals on a standard diet (C), animals fed an isoenergetic low-protein diet (LPD), animals subjected to acute APAP-induced toxicity (APAP), and animals fed a low-protein diet followed by APAP-induced toxicity (LPD/APAP). Values are presented as mean ± standard error (SE) for 9 animals per group. Overall differences among groups were assessed using the Kruskal–Wallis test. Subsequently, pairwise post hoc comparisons were performed using Mann–Whitney U tests with Holm correction, and significant differences are indicated by * (*p* = 0.003) and ** (*p* = 0.004) for the most notable values.

The weights of the liver and kidneys were found to be altered under all treatment conditions (LPD, APAP, LPD/APAP), as shown in Table 2. The low-protein diet (LPD) led to a marked reduction in both liver and kidney weights, approximately 30%, which was expected due to the overall decrease in body weight observed in this group. In the APAP group, animals exposed to toxic doses of APAP exhibited an increase of up to 20% in the relative mass of the liver and kidneys. In the LPD/APAP group, despite a 20% reduction in total body weight, the relative liver and kidney weights were significantly increased by 18.7 ± 1.9% and 15.3 ± 1.4%, respectively (Table 2, Figure 1). This was accompanied by visible signs of edema, resulting from inflammation and tissue damage, including fluid retention and immune cell infiltration. As the objective of the present study was the biochemical analysis of damaged kidney cells, detailed histopathological analysis was beyond the scope of this work. Such histological features were noted but not assessed systematically and will be examined in detail in a separate investigation.

The increases in the parameters reported in Table 2 are consistent with persistent edema observed in the APAP and LPD/APAP groups, where liver and kidney relative weights were also increased (Figure 1) with significances calculated: overall differences among groups were assessed using a Kruskal–Wallis test (H(3) = 24.07, p = 2.4 × 10^−5^ in Figure 1A and H(3) = 34.07, p = 1.9 × 10^−7^ in Figure 1B). Pairwise post hoc comparisons were performed using Mann–Whitney U tests with Holm correction. For relative liver weight (Figure 1A) control did not differ from LPD, and APAP did not differ from LPD/APAP, whereas both APAP and LPD/APAP were significantly higher than Control and LPD (adjusted *p* < 0.01). For relative kidney weight (Figure 1B) control did not differ from LPD, and APAP did not differ from LPD/APAP, whereas both APAP and LPD/APAP were significantly higher than Control and LPD (adjusted *p* < 0.01). The exact statistical parameters and *p* values are reported in Supplemental Table S3. Only the most important significant differences are reported in Figure 1 to avoid image overload.

### 3.3. Impact of Experimental Conditions on Adenine Nucleotide Balance

**Figure 2:**
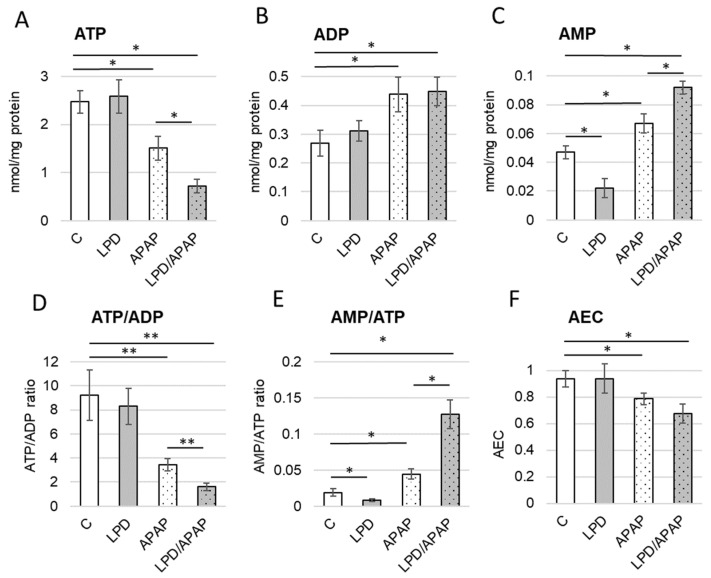
Low-protein diet and APAP-induced toxicity alter adenylate nucleotide content in kidney cells. The concentrations of ATP (**A**), ADP (**B**), AMP (**C**) were measured in cytosol form kidney cells isolated from rats in four experimental groups: control animals on a standard diet (column C), animals fed an isoenergetic low-protein diet (LPD), animals subjected to acute APAP-induced toxicity (APAP), and animals fed a low-protein diet followed by APAP-induced toxicity (LPD/APAP). Calculated ATP/ADP (**D**) and AMP/ATP (**E**) ratios, as well as the Adenylate Energy Charge (AEC) (**F**), are presented for the same animal groups. Values are presented as mean ± standard error (SE) for 9 animals per group. Overall differences among groups were assessed using the Kruskal–Wallis test. Subsequently, pairwise post hoc comparisons were performed using Mann–Whitney U tests with Holm correction, and significant differences are indicated by * (*p* = 0.002) and ** (*p* = 0.003) for the most notable values.

Following the observation of effects at the organ level, we assessed the energy status of kidney cells by measuring the concentrations of adenine nucleotides AMP, ADP, and, most importantly, ATP. The results are presented in Figure 2 and significances of differences were calculated: overall differences among groups were assessed using a Kruskal–Wallis test: (H(3) = 29.74, p = 1.6 × 10^−6^ in Figure 2A; H(3) = 25.28, p = 1.35 × 10^−5^ in Figure 2B; H(3) = 32.93, p = 3.3 × 10^−7^ in Figure 2C; H(3) = 29.74, p = 1.6 × 10^−6^ in Figure 2D; H(3) = 32.86, p = 3.4 × 10^−7^ in Figure 2E; H(3) = 27.82, p = 4.0 × 10^−6^ in Figure 2F. Pairwise post hoc comparisons were performed using Mann–Whitney U tests with Holm correction. For ATP (Figure 2A), no difference was observed between control and LPD, whereas APAP and LPD/APAP were significantly lower than control and LPD (*p* = 0.002). Additionally, LPD/APAP was significantly lower than APAP (*p* = 0.002). For ADP (Figure 2B), no difference was observed between control and LPD, whereas APAP and LPD/APAP were significantly higher than control and LPD (*p* = 0.002). No difference was observed between APAP and LPD/APAP. For AMP (Figure 2C), all groups differed significantly from each other (*p* = 0.002). For ATP/ADP ratio (Figure 2D), no difference between C and LPD was observed, whereas APAP and LPD/APAP were significantly lower than both C and LPD (*p* = 0.003). LPD/APAP was also significantly lower than APAP (*p* = 0.003). For AMP/ATP ratio (Figure 2E) all groups differed significantly from each other (*p* = 0.002). For AEC (Figure 2F), no difference between C and LPD was observed, whereas APAP and LPD/APAP were significantly lower than both C and LPD (*p* = 0.0023). No significant difference was observed between APAP and LPD/APAP. All exact statistical parameters and *p*-values are reported in Supplemental Table S4. To avoid visual clutter, only the most important significant differences are highlighted in Figure 2.

Under the toxicity induction in APAP group, the ATP content in rat kidney cells significantly decreased by 40.0 ± 7.5% (Figure 2A), accompanied by a significant increase in ADP by 63.7 ± 16.5% (Figure 2B) and AMP by 42.6 ± 13.7% (Figure 2C), compared to control values. This depletion of the ATP pool likely reflects enhanced ATP hydrolysis or impaired resynthesis of ATP. The most pronounced changes in adenine nucleotide content were observed in rats with APAP-induced toxicity that consumed a low-protein diet (LPD/APAP group). In the cells of these animals, ATP levels dropped to critically low values, decreasing by 71.1 ± 8.2%, while ADP levels remained comparable to those in the APAP group. In contrast, AMP levels nearly doubled (+95.8 ± 8.7%) compared to the control group (Figure 2C). Notably, in the LPD group (without APAP), significant changes were observed only in AMP levels, which decreased by 53.2 ± 6.4%.

The severity of the energetic imbalance is also evident from the ATP/ADP ratio, a widely used parameter of cellular energy status. While the ratio remained high in both the control and LPD groups, it decreased significantly by 2.7 ± 0.4-fold and 5.75 ± 1.2-fold to moderately low levels in the APAP and LPD/APAP groups, respectively (Figure 2D). Notably, the LPD/APAP group differed significantly from the APAP group, indicating the combined impact of the two damaging factors. The AMP/ATP ratio, one of the most sensitive indicators of cellular energy stress and known to increase under energy imbalance, also rose markedly. It increased 2.4 ± 0.4-fold in the APAP group and 7.1 ± 1.3-fold in the LPD/APAP group (Figure 2E). Although the magnitude of change in the intoxicated groups was not as large as expected and the LPD group even showed a decrease in the AMP/ATP ratio (−55.3 ± 4.1%, Figure 2E), the increase observed in the LPD/APAP group was the strongest. Moreover, the difference between the LPD/APAP and APAP groups was statistically significant for this parameter (Figure 2E).

The AEC an integrated indicator of cellular metabolic energy based on ATP, ADP, and AMP levels, remained unchanged in the LPD group. In contrast, it decreased significantly, though not drastically, in the APAP and LPD/APAP groups, by 17.2 ± 2.1% and 29.1 ± 4.2%, respectively (Figure 2F).

### 3.4. Alterations in Enzymes Involved in Energy Metabolism

**Figure 3:**
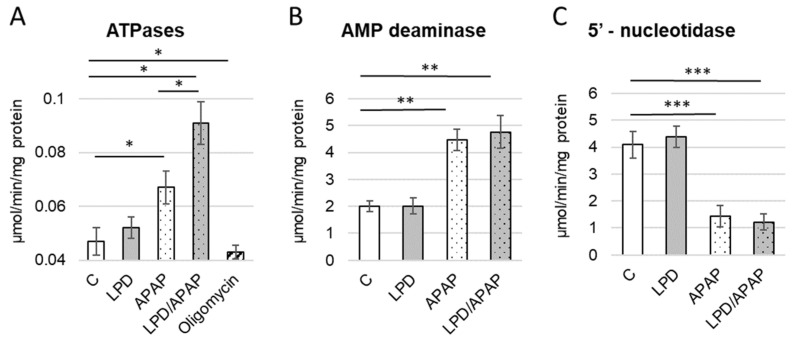
Low-protein diet and APAP-induced toxicity alter the activity of key energy-related enzymes in kidney. The activities of mitochondrial ATPases (**A**), cytosolic AMP deaminase (**B**), and cytosolic 5′-nucleotidase (**C**) were measured in kidney from rats in four experimental groups: control animals on a standard diet (column C), animals fed an isoenergetic low-protein diet (LPD), animals subjected to acute APAP-induced toxicity (APAP), and animals fed a low-protein diet followed by APAP-induced toxicity (LPD/APAP). Oligomycin was used to inhibit the activity of FoF_1_-ATPase. Values are presented as mean ± standard error (SE) for 9 animals per group. Overall differences among groups were assessed using the Kruskal–Wallis test. Subsequently, pairwise post hoc comparisons were performed using Mann–Whitney U tests with Holm correction, and significant differences are indicated by * (*p* = 0.0037), ** (*p* = 0.0023) and *** (*p* = 0.0024) for the most notable values.

Following the observed imbalance in adenine nucleotide levels, and considering that maintenance of the adenylate pool depends on a dynamic balance between de novo synthesis, resynthesis, and catabolism to end products, we next evaluated the activity of enzymes involved in this regulatory network. Specifically, we measured the activities of mitochondrial ATPases, cytosolic AMP deaminase, and cytosolic 5′-nucleotidase. The results are presented in Figure 3 and statistical analysis in Table S5. Statistical analysis was performed: overall differences among groups were assessed using a Kruskal–Wallis test: (H(4) = 40.39, p = 3.6 × 10^−8^ in Figure 3A; H(3) = 26.45, p = 7.7 × 10^−6^ in Figure 3B; H(3) = 26.93, p = 6.1 × 10^−6^ in Figure 3C. Post hoc pairwise comparisons using Mann–Whitney U tests with Holm correction indicated significant differences among all groups (adjusted *p* = 0.0037) for Figure 3A. Test for Figure 3B showed no difference between control and LPD, whereas APAP and LPD/APAP were significantly higher than both control and LPD (*p* = 0.0023). No significant difference was observed between APAP and LPD/APAP. Statistical tests for Figure 3C showed no difference between control and LPD, whereas APAP and LPD/APAP were significantly lower than both control and LPD (*p* = 0.0024). No significant difference was observed between APAP and LPD/APAP.

Measured in isolated intact mitochondria, ATPase activity is expected to be low, especially in healthy control animals. Indeed, compared with low basal activity in control group, we observed a significantly increased activation of mitochondrial ATPases enzymes in both the APAP and LPD/APAP groups, by 42.6 ± 5.3% and 93.6 ± 8.9%, respectively, with the strongest effect in the LPD/APAP group (Figure 3A). The increase in ATPase activity in the LPD/APAP group was also significantly higher than in the APAP group alone. Importantly, ATP levels were reduced in the same groups (Figure 2A), suggesting, for example, a functional shift in the FoF_1_-ATPase from ATP synthesis to ATP hydrolysis. The inhibition of ATPase activity by oligomycin (Figure 3A) indicates that the observed activity is primarily attributable to the FoF_1_-ATPase.

The activity of AMP deaminase was significantly increased vs. control in both the APAP and LPD/APAP groups by 2.2 ± 0.3-fold and 2.4 ± 0.4-fold, respectively (Figure 3B). In contrast, the activity of 5′-nucleotidase was markedly decreased in these groups by 2.9 ± 0.4-fold and 3.4 ± 0.3-fold, respectively (Figure 3C). The parallel partial increase in AMP and ADP levels observed in these cells (Figure 2B,C) will be addressed in detail in the Discussion section.

Notably, in the LPD group, no significant changes in the activity of any of the three enzymes were observed compared to the control group. This suggests that enzyme regulation in diet-only animals is robust, but disrupted under additional strong perturbations.

### 3.5. Impact of Toxic APAP Doses and Low-Protein Diet on Lipid Peroxidation and Protein Damage

**Figure 4:**
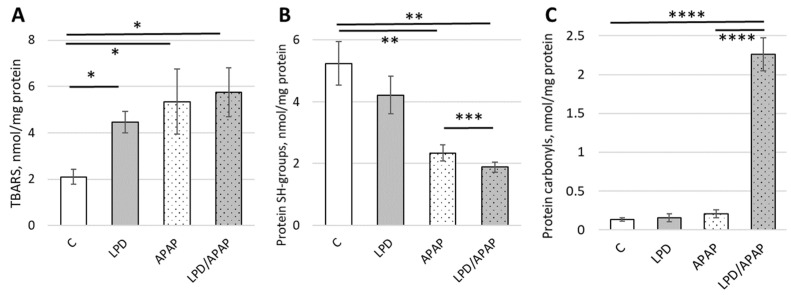
Low-protein diet and APAP-induced toxicity induce lipid peroxidation and protein damage in the mitochondrial fraction of kidney cells. The levels of thiobarbituric acid-reactive substances (TBARS, (**A**)), protein oxidation markers as protein SH-groups (**B**) and protein carbonils (**C**) were measured in kidney cells isolated form rats in four experimental groups: control animals on a standard diet (column C), animals fed an isoenergetic low-protein diet (LPD), animals subjected to acute APAP-induced toxicity (APAP), and animals fed a low-protein diet followed by APAP-induced toxicity (LPD/APAP). Values are presented as mean ± standard deviation (SD). Overall differences among groups were assessed using the Kruskal–Wallis test. Subsequently, pairwise post hoc comparisons were performed using Mann–Whitney U tests with Holm correction (Table S6), and significant differences are indicated by * (*p* = 0.0023), ** (*p* = 0.0024), *** (*p* = 0.0019) and **** (*p* = 0.0021) for the most notable values.

To understand the type of damage that could lead to enzyme dysregulation, we tested lipid peroxidation products and oxidative protein damage. Lipid peroxidation, measured as thiobarbituric acid-reactive substances (TBARS), was significantly increased over basal levels: 2.12 ± 0.41, 2.55 ± 0.78, and 2.74 ± 0.63 times for LPD, APAP, and LPD/APAP groups, respectively (Figure 4A). Overall differences among groups were assessed using a Kruskal–Wallis test: (H(3) = 24.52, p = 1.94 × 10^−5^). Control was significantly (*p* = 0.0023) lower than all other groups. LPD/APAP was significantly higher than LPD (*p* = 0.024), whereas no significant differences were observed between LPD and APAP or between APAP and LPD/APAP.

Since lipid peroxidation is triggered by oxidative stress, both ROS and lipid peroxidation products are expected to damage proteins by covalently reacting with protein SH groups. Overall differences among groups were significate (H(3) = 30.06, p = 1.34 × 10^−6^). Post hoc pairwise comparisons showed significant differences between all groups (adjusted *p* < 0.012). The quantity of protein unmodified SH groups decreased significantly in APAP and LPD/APAP groups by 2.24 ± 0.54 and 2.79 ± 0.65 times, respectively. The impact of APAP on diet animals was significant: the difference between APAP and LPD/APAP showed an 18.3 ± 2.8% decrease. The diet-only LPD group showed a reduction in protein SH groups (Figure 4B).

Another, harsher and more damaging covalent protein modification, protein carbonylation, was quantified. Relatively low baseline levels were observed in the control group of animals, as well as in the LPD and APAP groups. Under both damaging conditions in the LPD/APAP group, the level of protein carbonyls in kidney cells increased dramatically, rising 17.4 ± 3.2-fold, indicating a strong potential synergism or interference between the treatments. A Kruskal–Wallis test revealed a significant difference among groups (H(3) = 26.94, p = 6.0 × 10^−6^). LPD/APAP was significantly higher than all other groups (*p* = 0.0021), while APAP was significantly higher than C (*p* = 0.0068). No significant differences were observed between C and LPD or between LPD and APAP.

## 4. Discussion

Investigating the interplay between acetaminophen-induced hepatotoxicity and dietary protein deficiency is critically important, as these conditions may synergistically impair either hepatic or renal function. This combined impact may intensify oxidative stress and disrupt detoxification pathways. Understanding these interactions is essential for clarifying how energy homeostasis is compromised and for identifying potential targets for therapeutic and nutritional interventions.

It is known that dietary protein deficiency markedly affects somatic growth and organ development in animal models. In this study, protein restriction produced a behavioral change, growth-retardation phenotype, with final body weights approximately 20% lower than those of animals fed a complete diet (Table 1 and Table 2). Animals on the low-protein regimen exhibited significant reductions in absolute liver and kidney weights; however, when normalized to body weight, relative organ weights did not differ significantly from controls (Table 2). In contrast, animals administered toxic doses of APAP showed a significant increase in the relative weights of the liver and kidneys. This enlargement reflects early pathophysiological alterations, including cellular swelling and metabolic dysregulation associated with toxic injury. Histopathological examination revealed organ edema, a typical response to inflammation and tissue injury. This edema is attributable to enhanced vascular permeability, which promotes fluid accumulation and subsequent infiltration of immune cells, ultimately compromising kidney functionality.

We extended our study to the cellular and subcellular levels to investigate alterations in energy metabolism at the molecular level, aiming to better understand the mechanisms underlying the observed tissue injury. In the kidneys, the toxic APAP metabolite NAPQI, generated through CYP2E1-mediated biotransformation of APAP, initiates a cascade of damaging events characterized by glutathione depletion, formation of protein adducts, and activation of multiple pathological pathways, including mitochondrial impairment, which contribute to nephrotoxicity [42,43]. It has been described that toxic APAP exposure promotes mitochondrial protein release through activation of the mitochondrial permeability transition pore (mPTP) [44,45]. Although the mPTP is typically characterized as a single mechanistic entity, its opening likely occurs at multiple sites across the inner mitochondrial membrane. Such distributed pore formation could collectively disrupt membrane integrity, increase permeability to ATP, and strongly impair or reverse ATP synthase function. These combined effects may diminish the mitochondrial ATP pool which we have observed here (Figure 2). For this reason, we examined ATPases’ activity in the present study. We performed a protocol for the extraction of intact mitochondria. ATP was added to the assay, and under normal conditions, ATP should not enter intact mitochondria and therefore should not be hydrolyzed. In the control group, only a very low (background) amount of ATP was hydrolyzed. However, in the APAP and LPD/APAP groups, mitochondrial integrity was significantly compromised by APAP toxicity. Consequently, we observed a markedly higher level of ATP hydrolysis, indicating that ATP entered the damaged mitochondria and was hydrolyzed, most likely by ATP synthase (Complex V, FoF1-ATPase), which under toxic conditions operated in reverse as an ATPase. This interpretation was further supported by the effect of oligomycin, a specific ATP-synthase inhibitor, which blocked the enzyme activity (Figure 4A). Additionally, APAP intoxication has been reported before to compromise the function of respiratory chain enzymes NADH:ubiquinone oxidoreductase and succinate:ubiquinone oxidoreductase [26], which also could contribute to reduced ATP production (Figure 2).

Regarding the AMP imbalance observed in this study (Figure 2), several considerations can be made. AMP is recognized as an allosteric activator of AMP-activated protein kinase (AMPK, EC 2.7.11.31), a key regulator of cellular energy homeostasis that stimulates catabolic pathways to generate ATP during energy-deficient states while inhibiting anabolic processes [46]. In response to ATP depletion (Figure 2) and increased oxidative stress (Figure 4), the observed elevation of AMP in kidney cells likely enhances AMPK activity, which is essential for maintaining energy balance and redirecting metabolic fluxes. Moreover, in our model, AMPK activation may enhance cellular antioxidant defenses through the NRF2/HO-1 signaling pathway [47], thereby attempting to provide an adaptive response to the oxidative stress induced by APAP toxicity. However, despite these potential protective mechanisms activated in the studied cells in response to damaging factors, the ATP/ADP, AMP/ATP, and AEC ratios remained imbalanced, indicating an insufficient restoration of energy homeostasis. Further investigation of AMPK and the NRF2/HO-1 pathway represents a potential future direction of this work.

Regarding enzymes involved in AMP catabolism, we found that in animals with both APAP-induced toxicity and protein deficiency (LPD/APAP group), the activities of AMP deaminase (Figure 3B) and 5′-nucleotidase (Figure 3C) did not differ significantly from those in the APAP-only group, where the effect was markedly strong vs. control. This suggests that toxic APAP-products exert a dominant effect on adenine nucleotide catabolism by ROS induction and lipid peroxidation. The observed increase in AMP levels under APAP intoxication (Figure 2) may contribute to the modulation of enzymatic activities of both enzymes (Figure 3B,C). AMP deaminase, the rate-limiting enzyme of AMP degradation, catalyzes the conversion of AMP to IMP and ammonia, while 5′-nucleotidase hydrolyses AMP or IMP to their respective nucleosides and inorganic phosphate [13,17,48]. Our analysis revealed that, in the cytosolic fraction of rat kidneys exposed to APAP, AMP deaminase activity increased more than twofold (Figure 3B), whereas 5′-nucleotidase activity decreased nearly threefold (Figure 3C). These findings suggest a metabolic shift favoring AMP deamination over nucleotide hydrolysis under APAP toxicity, potentially serving as a regulatory mechanism to sustain energy homeostasis. IMP, the product of AMP deaminase, is a central intermediate in purine metabolism and can be converted into AMP, inosine, or guanosine monophosphate as part of nucleotide salvage and interconversion pathways. This may represent a compensatory response to maintain nucleotide balance. Furthermore, inosine derived from IMP has been reported to play a critical role in activating antioxidant defenses and intracellular signaling. Inosine regulates the expression of several crucial proteins involved in oxidative stress response and signaling pathways, including superoxide dismutase (SOD), sirtuins (SIRT), IκB-α, and inducible nitric oxide synthase (iNOS) [49,50]. Thus, in our experimental model, IMP and inosine may act as signaling molecules contributing to antioxidant defense and cellular adaptation under APAP-induced stress.

A likely explanation for the decreased 5′-nucleotidase activity under APAP toxicity is the enhancement of intracellular oxidative stress, as reactive oxygen species are known to irreversibly inactivate this enzyme [51]. In our study, we measured lipid peroxidation using TBARS and detected elevated levels in all experimental groups, with the highest values observed in the APAP and LPD/APAP groups. Consistent with the presence of lipid peroxidation products, additional indicators of protein damage were also observed. Protein SH groups were significantly reduced, indicating oxidative and/or lipoperoxidative modification of thiol-containing proteins. Protein carbonyls were strongly increased in the LPD/APAP group, reflecting the formation of covalent protein conjugates that arise either from direct amino acid oxidation or from adduction by carbonyl-containing lipid peroxidation or glycoxidation (sugar oxidation) products, all of which may compromise protein function. These findings align with previous reports describing APAP-induced oxidative stress [52] and lipid peroxidation as a key mechanism contributing to liver injury [53]. Among the major lipid peroxidation products is 4-HNE, which covalently modifies proteins (protein lipoxidation) [22]. At low concentrations, 4-HNE can exert regulatory signaling functions [54,55,56], whereas at higher concentrations it exhibits protein- and DNA-damaging properties, and elevated 4-HNE levels have been associated with the impairments in a variety of pathological conditions [54,55,56,57,58,59]. In the literature, numerous functionally important proteins, including enzymes and receptors, have been shown to undergo 4-HNE adduction with significant functional consequences [22,54,60,61,62]. In the context of acetaminophen-induced oxidative stress, mitochondrial proteins such as sarcosine dehydrogenase and carbamoyl phosphate synthase-1 were reported to leak into the cytosol and undergo 4-HNE adduction [45], indicating substantial 4-HNE formation and suggesting that additional functional proteins may likewise be affected. Importantly, kidney cells have not been thoroughly investigated with respect to lipid peroxidation as a consequence of APAP toxicity, highlighting the relevance of the present findings.

As another consequence of the observed effects, increased AMP deaminase activity may stimulate the activity of key glycolytic enzymes, such as phosphofructokinase and pyruvate kinase. Under APAP-induced nephrotoxicity, this may represent a compensatory mechanism and an adaptive metabolic response aimed at preserving cellular energy homeostasis [12,63].

Potential targets for therapeutic and nutritional interventions could include strategies to restore mitochondrial function. Therapies that stabilize mitochondrial membranes or prevent mitochondrial permeability transition may be beneficial in preserving energy production and preventing cell damage. Regulation of energy metabolism could also be explored further. The AMP-activated protein kinase (AMPK) pathway plays a central role in sensing cellular energy status, and targeting this pathway might help cells adapt to energy stress. Additionally, glycolytic enzymes such as phosphofructokinase and pyruvate kinase, which respond to AMP levels, may be involved in metabolic adaptation and could serve as potential intervention points [63]. It may also be valuable to evaluate whether inhibiting AMP deaminase could help preserve AMP and support ATP regeneration [14,64].

Interventions aimed at reducing oxidative stress are another important area. Antioxidants such as N-acetylcysteine (NAC), vitamins, and glutathione-boosting compounds may help mitigate cellular damage, with extracellular vesicle-mediated delivery offering an additional delivery route [65,66]. Activating protective pathways such as Nrf2 may further enhance cellular defense mechanisms [67,68,69,70]. The development of new analgesic substances or pharmaceuticals that do not cause such severe oxidative damage is a rapidly evolving research field [71,72,73]. Nutritional support is especially important, as protein deficiency worsens the effects of APAP toxicity. Ensuring adequate protein intake or supplementing with specific amino acids, such as cysteine and methionine, could support detoxification processes and promote tissue repair.

Taken together, the detected changes in adenine nucleotide content under the combined effect of APAP toxicity and protein deficiency indicate the onset of energy deficiency, which could adversely affect kidney function. These findings are important for understanding how energy homeostasis is disrupted under such metabolic stress and for identifying potential therapeutic targets. Further investigation into the molecular mechanisms linking oxidative stress, mitochondrial functionality, and energy metabolism is warranted to fully elucidate the compensatory response.

## 5. Conclusions

This study demonstrates that acetaminophen (APAP) intoxication, especially when combined with dietary protein deficiency, severely disrupts renal energy metabolism and promotes oxidative stress. APAP overdose alone reduced ATP levels and increased AMP and ATPase activity in kidney mitochondria, indicating impaired energy balance. These effects were markedly amplified under low-protein nutrition, which also intensified lipid peroxidation and oxidative protein damage. Together, these findings suggest that nutritional status critically modulates drug-induced nephrotoxicity. Importantly, the observed oxidative damage highlights the potential role of antioxidant strategies in mitigating mitochondrial dysfunction and protecting kidney integrity under conditions of metabolic stress.

## Figures and Tables

**Table 1:** Clinical scoring of physiological and behavioral status in rats on day 129 of life in the control group (Control), the isoenergetic low-protein diet group (LPD), the group subjected to acute toxic injury induced by APAP (APAP), and the group exposed to both a low-protein diet and APAP toxicity (LPD/APAP). Scores were assigned as follows: 0 = normal physiological status; 1 = mild impairment; 2 = moderate impairment; 3 = severe impairment. Data are expressed as median (interquartile range) for 9 animals per group. Overall differences among groups were assessed using a Kruskal–Wallis test: H(3) parameter and significance p are presented for each row. Pairwise post hoc comparisons were performed using two-sided Mann–Whitney U tests with Holm correction for multiple testing (Supplemental Table S1). Groups not sharing the same letter (a, b, c, d) differ significantly (adjusted *p* < 0.05).

	Control	LPD	APAP	LPD/APAP	H(3), p
Apathy	0 (0–0) a	1 (0–2) b	2 (1–2) b	3 (2–3) c	23.61, p = 2.32 × 10^−5^
Fur Condition (Dullness/Loss))	0 (0–0) a	2 (1–2) b	1 (1–1) c	2 (2–2) d	28.40, p = 2.99 × 10^−6^
Motor Coordination	0 (0–0) a	0 (0–1) b	1 (1–2) c	2 (2–2) d	27.85, p = 3.91 × 10^−6^
General Clinical Score (sum of individual scores)	0 (0–0) a	3 (3–4) b	5 (3–5) c	7 (6–7) d	30.78, p = 9.47 × 10^−7^

**Table 2:** Effects of a low-protein diet and APAP-induced toxicity on liver and kidney weights in treated rats in the control group (Control), the isoenergetic low-protein diet group (LPD), the group subjected to acute toxic injury induced by APAP (APAP), and the group exposed to both a low-protein diet and APAP toxicity (LPD/APAP). Morphological parameters assessed include: body weight on day 129 of life, absolute and relative liver weight, and absolute and relative kidney weight. Values are presented as mean ± standard error (SE) for 9 animals per group in first line and as median (interquartile range) in second line. Overall differences among groups were assessed using a Kruskal–Wallis test: H(3) parameter and significance p are presented for each row. Pairwise post hoc comparisons were performed using two-sided Mann–Whitney U tests with Holm correction for multiple testing (Supplemental Table S2). Groups not sharing the same letter (a, b, c, d) differ significantly (adjusted *p* < 0.05).

	Control	LPD	APAP	LPD/APAP	H(3), p
Final body weight (g)	192 ± 7 193 (186–200) b	155 ± 7 154 (149–170) a	186 ± 5 185 (180–190) b	150 ± 5 155 (148–158) a	24.79, 1.71 × 10^−5^
Absolute liver weight (g)	6.77 ± 1.15 6.20 (5.90–7.92) b	4.96 ± 0.74 4.50 (4.20–5.40) a	8.27 ± 1.21 7.50 (7.10–9.40) c	6.43 ± 0.59 6.20 (5.90–7.02) b	24.67, 1.8 × 10^−5^
Relative liver weight (mg/g body weight)	35.63 ± 0.43 35.50 (35.30–36.00) a	32.42 ± 0.24 32.30 (32.20–32.60) b	43.99 ± 0.55 43.80 (43.50–44.54) c	42.3 ± 0.37 42.10 (41.95–42.67) d	32.84, 3.48 × 10^−7^
Absolute kidney weight (g)	1.53 ± 0.18 1.40 (1.38–1.70) b	1.11 ± 0.07 1.07 (1.05–1.16) a	1.63 ± 0.09 1.58 (1.55–1.71) c	1.41 ± 0.11 1.35 (1.32–1.50) b	25.62, 1.14 × 10^−5^
Relative kidney weight (mg/g body weight)	8.05 ± 0.12 7.99 (7.95–8.15) b	7.25 ± 0.68 6.90 (6.70–7.90) a	8.67 ± 0,5 8.30 (8.20–9.20) c	9.28 ± 1.02 8.60 (8.40–10.20) c	25.84, 1.03 × 10^−5^

## Data Availability

The original contributions presented in the study are included in the article. Further inquiries can be directed to the corresponding authors.

## Supplementary Materials

The following supporting information can be downloaded at: https://www.mdpi.com/article/10.3390/antiox15010105/s1, Table S1: Pairwise post-hoc comparisons of groups after Kruskal–Wallis test reported in Table 1. Table S2: Pairwise post-hoc comparisons of groups after Kruskal–Wallis test reported in Table 2. Table S3: Post-hoc pairwise comparisons between groups for Figure. Table S4: Post-hoc pairwise comparisons between groups for Figure 2. Table S5: Post-hoc pairwise comparisons between groups for Figure 3. Table S6. Post-hoc pairwise comparisons between groups for Figure 4.
