# Supplementary material for: Alterations in Adenylate Nucleotide Metabolism and Associated Lipid Peroxidation and Protein Oxidative Damage in Rat Kidneys Under Combined Acetaminophen Toxicity and Protein Deficiency
Source: Antioxidants (Basel). 2026 Jan 13;15(1):105. doi: 10.3390/antiox15010105 (PMC12837607; doi:10.3390/antiox15010105)
Supplement: Supplementary file 1 [file antioxidants-15-00105-s001.zip › antioxidants-4067956-supplementary.pdf]

Supplemental material for paper "Alterations in Adenylate Nucleotide Metabolism and Associated Lipid Peroxidation and Protein Oxidative Damage in Rat Kidneys Under Combined Acetaminophen Toxicity and Protein Deficiency" by Voloshchuk et al.

Table S1. Pairwise post-hoc comparisons of groups after Kruskal–Wallis test reported in Table 1. Clinical scoring of physiological and behavioral status was assisted for group on day 129 of life in the control group (Control), the isoenergetic low-protein diet group (LPD), the group subjected to acute toxic injury induced by APAP (APAP), and the group exposed to both a low-protein diet and APAP toxicity (LPD/APAP). Scores were assigned as follows: 0 = normal physiological status; 1 = mild impairment; 2 = moderate impairment; 3 = severe impairment. Data are expressed as median (interquartile range) for 9 animals per group and reported in Table 1. Overall differences among groups were assessed using a Kruskal–Wallis test: H(3) and significance p are presented for each row in Table 1. Pairwise post-hoc comparisons were performed using two-sided Mann–Whitney U tests with Holm correction for multiple testing and reported here. "U" means "value U di Mann-Whitney", "ns" means the difference is non-significant.

Comparison	U	Adjusted p-value (Holm)
Apathy		
Control vs LPD	9.0	0.0067
Control vs APAP	4.5	0.0024
Control vs LPD/APAP	0.0	<0.001
LPD vs APAP	30.5	0.36 (ns)
LPD vs LPD/APAP	9.5	0.015
APAP vs LPD/APAP	14.0	0.029
Fur Condition		
C vs LPD	0.0	0.00061
C vs APAP	4.5	0.00144
C vs LPD/APAP	0.0	0.00056
LPD vs APAP	60.5	0.04486
LPD vs LPD/APAP	17.5	0.03409
APAP vs LPD/APAP	3.5	0.00147
Motor Coordination		
C vs LPD	22.5	0.064
C vs APAP	0.0	0.00069
C vs LPD/APAP	0.0	0.00069
LPD vs APAP	10.0	0.0119
LPD vs LPD/APAP	2.0	0.00189

APAP vs LPD/APAP	18.5	0.064
General Clinical Score		
C vs LPD	0.0	0.000775
C vs APAP	0.0	0.000775
C vs LPD/APAP	0.0	0.000775
LPD vs APAP	19.5	0.0565 (ns)
LPD vs LPD/APAP	0.5	0.00126
APAP vs LPD/APAP	2.5	0.00137

Table S2. Pairwise post-hoc comparisons of groups after Kruskal–Wallis test reported in Table 2. Effects of a low-protein diet and APAP-induced toxicity on liver and kidney weights in treated rats in the control group (Control), the isoenergetic low-protein diet group (LPD), the group subjected to acute toxic injury induced by APAP (APAP), and the group exposed to both a low-protein diet and APAP toxicity (LPD/APAP). Morphological parameters assessed include: final body weight on day 129 of life, absolute and relative liver weight, and absolute and relative kidney weight. Data are expressed as means and median (interquartile range) for 9 animals per group and reported in Table 2. Overall differences among groups were assessed using a Kruskal–Wallis test: H(3) and significance p are presented for each row in Table 2. Pairwise post-hoc comparisons were performed using two-sided Mann–Whitney U tests with Holm correction for multiple testing and reported here. “U” means “value U di Mann-Whitney”, “ns” means the difference is non-significant.

Comparison	U	Adjusted p-value (Holm)
Final body weight		
Control vs LPD	78.0	0.00038
Control vs APAP	61.0	0.07250
Control vs LPD/APAP	81.0	0.00038
LPD vs APAP	5.5	0.00038
LPD vs LPD/APAP	43.0	0.85924
APAP vs LPD/APAP	81.0	0.00038
Absolute liver weight		
Control vs LPD	81.0	0.00038
Control vs APAP	45.0	0.00302
Control vs LPD/APAP	61.0	0.07250
LPD vs APAP	0.0	0.00038

LPD vs LPD/APAP	5.0	0.00038
APAP vs LPD/APAP	81.0	0.00038
Relative liver weight		
Control vs LPD	81.0	0.00041
Control vs APAP	81.0	0.00041
Control vs LPD/APAP	0.0	0.00041
LPD vs APAP	81.0	0.00041
LPD vs LPD/APAP	0.0	0.00041
APAP vs LPD/APAP	81.0	0.00041
Absolute kidney weight		
Control vs LPD	81.0	0.00041
Control vs APAP	58.5	0.12189
Control vs LPD/APAP	56.0	0.18465
LPD vs APAP	81.0	0.00041
LPD vs LPD/APAP	0.0	0.00041
APAP vs LPD/APAP	78.0	0.00107
Relative kidney weight		
Control vs LPD	74.0	0.00353
Control vs APAP	73.5	0.00407
Control vs LPD/APAP	4.0	0.00148
LPD vs APAP	80.5	0.00048
LPD vs LPD/APAP	0.5	0.00048
APAP vs LPD/APAP	27.5	0.26920

Table S3. Post-hoc pairwise comparisons between groups for Figure 1. Overall differences among groups were assessed using a Kruskal–Wallis test: $H(3) = 24.07$, $p = 2.4 \times 10^{-5}$ in Figure 1, A for relative liver weight and $H(3)= 34.07$, $p = 1.9 \times 10^{-7}$ in Figure 1, B for relative kidney weight. Pairwise post-hoc comparisons were performed using Mann-Whitney U tests with Holm correction, reporting two-sided, Holm-adjusted p values.

Comparison	U	Adjusted p-value (Holm)
Relative liver weight		
Control vs LPD	63.0	0.053 (ns)
Control vs APAP	4.5	0.0033
Control vs LPD/APAP	4.5	0.0033

Comparison	U	Adjusted p-value (Holm)
Relative liver weight		
LPD vs APAP	3.0	0.0039
LPD vs LPD/APAP	3.0	0.0039
APAP vs LPD/APAP	44.5	0.75 (ns)
Relative kidney weight		
CTRL vs LPD	85.0	0.0043
CTRL vs APAP	0.0	0.00037
CTRL vs LPD/APAP	0.0	0.00037
LPD vs APAP	0.0	0.00068
LPD vs LPD/APAP	0.0	0.00068
APAP vs LPD/APAP	15.5	0.0094

Table S4. Post-hoc pairwise comparisons between groups for Figure 2. Overall differences among groups were assessed using a Kruskal–Wallis test: ($H(3) = 29.74$, $p = 1.6 \times 10^{-6}$ in Figure 2, A; $H(3) = 25.28$, $p = 1.35 \times 10^{-5}$ in Figure 2, B; $H(3) = 32.93$, $p = 3.3 \times 10^{-7}$ in Figure 2, C; $H(3) = 29.74$, $p = 1.6 \times 10^{-6}$ in Figure 2, D; $H(3) = 32.86$, $p = 3.4 \times 10^{-7}$ in Figure 2, E; $H(3) = 27.82$, $p = 4.0 \times 10^{-6}$ in Figure 2, F. Pairwise post-hoc comparisons were performed using Mann-Whitney U tests with Holm correction, reporting two-sided, Holm-adjusted p values.

Comparison	U	Adjusted p-value (Holm)
ATP		
Control vs LPD	33.5	0.56 (ns)
Control vs APAP	81.0	0.0024
Control vs LPD/APAP	81.0	0.0024
LPD vs APAP	81.0	0.0024
LPD vs LPD/APAP	81.0	0.0024
APAP vs LPD/APAP	81.0	0.0024
ADP		

C vs LPD	17.0	0.076 (ns)
C vs APAP	0.0	0.0023
C vs LPD/APAP	0.0	0.0023
LPD vs APAP	5.0	0.0057
LPD vs LPD/APAP	4.0	0.0057
APAP vs LPD/APAP	35.5	0.69 (ns)
AMP		
C vs LPD	81.0	0.0022
C vs APAP	0.0	0.0022
C vs LPD/APAP	0.0	0.0022
LPD vs APAP	0.0	0.0022
LPD vs LPD/APAP	0.0	0.0022
ATP/ADP		
C vs LPD	53.5	0.27 (ns)
C vs APAP	81.0	0.0025
C vs LPD/APAP	81.0	0.0025
LPD vs APAP	81.0	0.0025
LPD vs LPD/APAP	81.0	0.0025
APAP vs LPD/APAP	80.0	0.0025
AMP/ATP		
C vs LPD	81.0	0.0024
C vs APAP	0.0	0.0024
C vs LPD/APAP	0.0	0.0024
LPD vs APAP	0.0	0.0024
LPD vs LPD/APAP	0.0	0.0024
APAP vs LPD/APAP	0.0	0.0024

AEC		
C vs LPD	37.5	0.82 (ns)
C vs APAP	81.0	0.0023
C vs LPD/APAP	81.0	0.0023
LPD vs APAP	81.0	0.0023
LPD vs LPD/APAP	81.0	0.0023
APAP vs LPD/APAP	66.0	0.051 (ns)

Table S5. Post-hoc pairwise comparisons between groups for Figure 3. Overall differences among groups were assessed using a Kruskal–Wallis test (($H(4) = 40.39$, $p = 3.6 \times 10^{-8}$in Figure 3, A; $H(3) = 26.45$, $p = 7.7 \times 10^{-6}$ in Figure 3, B; $H(3) = 26.93$, $p = 6.1 \times 10^{-6}$ in Figure 3, C.). Pairwise post-hoc comparisons were performed using Mann-Whitney U tests with Holm correction, reporting two-sided, Holm-adjusted p values.

Comparison	U	Adjusted p-value (Holm)
ATPases		
C vs LPD	4.5	0.0037
C vs APAP	0.0	0.0037
C vs LPD/APAP	0.0	0.0037
C vs Oligomycin	64.5	0.0369
LPD vs APAP	0.5	0.0037
LPD vs LPD/APAP	0.0	0.0037
LPD vs Oligomycin	81.0	0.0037
APAP vs LPD/APAP	0.0	0.0037
APAP vs Oligomycin	81.0	0.0037
LPD/APAP vs Oligomycin	81.0	0.0037
AMP deaminase		
C vs LPD	36.0	1.00 (ns)
C vs APAP	0.0	0.0023

C vs LPD/APAP	0.0	0.0023
LPD vs APAP	0.0	0.0023
LPD vs LPD/APAP	0.0	0.0023
APAP vs LPD/APAP	36.0	1.00 (ns)
5′-nucleotidase		
C vs LPD	31.0	0.43 (ns)
C vs APAP	81.0	0.0024
C vs LPD/APAP	81.0	0.0024
LPD vs APAP	81.0	0.0024
LPD vs LPD/APAP	81.0	0.0024
APAP vs LPD/APAP	55.0	0.43 (ns)

Table S6. Post-hoc pairwise comparisons between groups for Figure 4. Overall differences among groups were assessed using a Kruskal–Wallis test (($H(3) = 24.52$, $p = 1.94 \times 10^{-5}$ for Figure 4, A; $H(3) = 30.06$, $p = 1.34 \times 10^{-6}$ for Figure 4, B; ($H(3) = 26.94$, $p = 6.0 \times 10^{-6}$ for Figure 4, C). Pairwise post-hoc comparisons were performed using Mann-Whitney U tests with Holm correction, reporting two-sided, Holm-adjusted p values.

Comparison	U	Adjusted p-value (Holm)
TBARS		
C vs LPD	0.0	0.0023
C vs APAP	0.0	0.0023
C vs LPD/APAP	0.0	0.0023
LPD vs APAP	16.5	0.075 (ns)
LPD vs LPD/APAP	10.0	0.024
APAP vs LPD/APAP	31.5	0.45 (ns)
Protein SH-groups		
C vs LPD	70.0	0.0119
C vs APAP	81.0	0.0024

Comparison	U	Adjusted p-value (Holm)
TBARS		
C vs LPD/APAP	81.0	0.0024
LPD vs APAP	81.0	0.0024
LPD vs LPD/APAP	81.0	0.0024
APAP vs LPD/APAP	72.0	0.0119
Protein carbonylation		
C vs LPD	15.5	0.054 (ns)
C vs APAP	6.0	0.0068
C vs LPD/APAP	0.0	0.0021
LPD vs APAP	17.5	0.054 (ns)
LPD vs LPD/APAP	0.0	0.0021
APAP vs LPD/APAP	0.0	0.0021
